# Proximity to Coast Is Linked to Climate Change Belief

**DOI:** 10.1371/journal.pone.0103180

**Published:** 2014-07-21

**Authors:** Taciano L. Milfont, Laurel Evans, Chris G. Sibley, Jan Ries, Andrew Cunningham

**Affiliations:** 1 School of Psychology, Victoria University of Wellington, Wellington, New Zealand; 2 Department of Psychology, University of Auckland, Auckland, New Zealand; 3 School of Geography, Environment and Earth Sciences, Victoria University of Wellington, Wellington, New Zealand; US Army Engineer Research and Development Center, United States of America

## Abstract

Psychologists have examined the many psychological barriers to both climate change belief and concern. One barrier is the belief that climate change is too uncertain, and likely to happen in distant places and times, to people unlike oneself. Related to this perceived psychological distance of climate change, studies have shown that direct experience of the effects of climate change increases climate change concern. The present study examined the relationship between physical proximity to the coastline and climate change belief, as proximity may be related to experiencing or anticipating the effects of climate change such as sea-level rise. We show, in a national probability sample of 5,815 New Zealanders, that people living in closer proximity to the shoreline expressed greater belief that climate change is real and greater support for government regulation of carbon emissions. This proximity effect held when adjusting for height above sea level and regional poverty. The model also included individual differences in respondents' sex, age, education, political orientation, and wealth. The results indicate that physical place plays a role in the psychological acceptance of climate change, perhaps because the effects of climate change become more concrete and local.

## Introduction

The vast majority of climate scientists (97–98%) agree with the reality and human causes of climate change [1]. However, it is clear that climate change poses a unique and profoundly complex problem, one that is not easily communicated. A study found that only 53% of the New Zealand public believe climate change is real and caused by humans, while 10% do not believe in climate change, 7% believe it is real but not caused by humans, and a large proportion (31%) remain undecided [2]. Surveys conducted in the U.S. report similar results, with three showing that around 63–69% of Americans believe global warming is happening [3]–[5] and another reporting that 82% of Americans believe climate change is happening [6]. Other Western countries also show relatively moderate levels of concern compared to climate scientists [7]. At the same time, support for action to reduce global warming is moderate to high: 64% of New Zealanders say that citizens should be exerting more effort toward combating climate change [8], and, when they were shown 19 possible government policy options to reduce emissions, New Zealanders showed more support than opposition for all, with majority support for 17 of the policy options [9]. Again similarly, 68% of the U.S. public supports medium to large-scale efforts to tackle climate change [10]. Altogether, New Zealanders, similarly to Americans, want action taken on climate change, but it is clear that the public is significantly less certain about the threat than the scientists.

Scientists have tried communicating facts as one avenue to bridge the gap between scientific evidence and public perceptions of global climate change. But this deficit model of communication, which assumes that the public merely needs to be informed or enlightened via the presentation of facts in order to perceive the existing risks, is now largely considered to be too simple [11]. Lack of information is indeed one barrier for public awareness of climate change risks, in concert with the distortion of information due to the disproportionate representation of contrarians in popular media [12], [13]. However, the idea of psychological barriers has been gaining traction: There are a whole host of potential attitudes, beliefs, and feelings that can hinder perceiving, understanding, and acting upon climate change. For example, the way people tend to conceive issues differs when the problem is only described to them, as opposed to experienced over time [14]. In one study, people's misconceptions about CO_2_ were significantly reduced when they participated in a climate change simulator, as opposed to merely receiving summary information about it [15]. Additionally, in the last decades psychologists have identified numerous other barriers [16]–[20], which include (but may not be limited to): uncertainty, skepticism, distrust in information sources, externalizing responsibility and blame, optimism bias, attention to other priorities, reluctance to change lifestyles, habit, fatalism, a “drop in the ocean” feeling, lack of perceived political, business, and/or industry action, ideological, political and religious beliefs, worry about free riders, social norms/expectations, lack of enabling initiatives, belief in technological salvation, and the perception that climate change is a distant threat.

In particular, this last barrier––psychological distance––manifests in feelings that the problem is too uncertain (likelihood distance), will occur far away (geographical distance), far in the future (temporal distance), and to people different from oneself (social distance) [10], [21], [22]. For example, the geographical distance of environmental problems, including global warming, is apparent in respondents from 26 countries, who overall indicated that problems are likely to affect distant areas more severely than local ones [23]. Notably, the countries surveyed were both economically and environmentally diverse: Their Gross National Products varied widely, and the countries also varied on environmental performance in five domains, such as the level of local environmental impact on humans. The perceived geographical distance was unrelated to these objective indicators of country wealth and environmental quality––that is, respondents from these countries tended to perceive global warming as affecting others more than themselves, even when they are poorer and/or more environmentally vulnerable than others. Self-reported psychological distance has also been shown to be associated with both lower climate change concern and diminished willingness to reduce energy use [22], suggesting that when people experience psychological distance, it negatively affects both their climate change concern and their willingness to take action.

The strength of those feelings of psychological distance from climate change may be influenced by people's perceptions and beliefs about the levels of risk, as well as by actual exposure to hazards [24]–[28]. For example, the perception of increased climate change risk to the self and family is linked to willingness to change behaviors [29]. Similarly, when people consider possible local adaptations to climate change—an action that likely increases thoughts about local risk—this also increases their willingness to take personal action to reduce emissions [30]. More directly, personal experience with climate change effects, such as flooding or extreme weather events, is related to greater climate change concern [31], [32], especially when the events are recognized as related to climate change [32]. Even experiencing a warmer-than-usual day increases climate change concern [33], [34]. In general, exposure to personal risk or awareness of greater personal risk increases concern and/or willingness to act.

Since prior experience of weather events is intrinsically related to the region in which one lives, geographic location is another factor with the strong possibility of affecting psychological distance to climate change. In particular, in the present paper we focus on proximity to the coastline. Although all types of regions will experience climate changes of varying levels of severity, an IPCC report suggests that, in addition to mountain settlements and megacities, coastal communities are particularly vulnerable to the impacts of climate change [35], possibly because they must bear the brunt of sea-level rise, changing wind patterns, and/or increasingly severe tropical storms [36]. Thus, coastal dwellers' experience of current climate change impacts may be increased, and this may in turn lower their psychological distance to climate change––that is, they may take warnings of climate change more seriously because they feel they are beginning to experience it themselves. Accordingly, some studies have already focused on perceptions of risk near the coastline. For example, Brody et al. [37] showed in a U.S. study that risk from climate change is perceived to be significantly lower for respondents located farther away from the coastline. Indeed, among the other geo-physical variables considered in this study (e.g., relative elevation, sea-level rise/inundation risk, temperature trend), distance to the coast had the strongest association with climate change risk perception. Further, in another national sample of U.S. residents, Brody et al. [29] found proximity to the coast to be significantly and positively related to willingness to reduce personal emissions.

These U.S. findings support the view that proximity to the coast is an important variable to consider when examining public risk perception and willingness to act in response to climate change. Given that no similar studies have been conducted outside of the U.S., we chose to examine the distance to coast effect in a national probability sample of a coastal nation: New Zealand. We measured climate change belief and support for government action to regulate emissions, two similar but distinct outcome variables compared to previous ones (i.e., risk perception and willingness to reduce personal emissions). In addition to the value of such a study for Australasian researchers and policymakers, we hope that an independent study, conducted on a different national sample, will have international relevance by adding confidence that proximity affects climate change attitudes in general. The findings could also have theoretical implication for the psychological distance of climate change.

## Methods

### Ethics Statement

This study analyzed data from the New Zealand Attitudes and Values Study (NZAVS), which is a larger longitudinal research project. The present article only focuses on the first wave of the NZAVS collected in 2009. The overall NZAVS project was approved by the University of Auckland Human Ethics Committee (IRB approved). The first phases of this longitudinal study were approved on 09-September-2009 for 3 years, reference number: 2009/336. Ethics approval for the study was re-approved by the University of Auckland Human Participants Ethics Committee on 17-February-2012 until 09-September-2015. Reference number: 6171. NZAVS data is hosted at the University of Auckland. Contact details are removed when the questionnaires are received. All data were de-identified before analyses were conducted. The de-identified data is available to appropriately qualified researchers upon request for the purposes of re-analysis. The syntax for the models reported in this paper can be found at http://www.psych.auckland.ac.nz/en/about/our-research/research-groups/new-zealand-attitudes-and-values-study/nzavs-information-for-researchers.html.

#### Sampling procedure and participant details

The NZAVS-09 questionnaire was posted to 40,500 New Zealanders randomly selected from the 2009 New Zealand electoral roll. Roughly 1.36% of all people registered to vote were contacted and invited to participate. The NZAVS-09 contained responses from 6,518 participants, with a response rate of 16.6% [38]. Complete data for all exogenous measures analyzed here were available for 5,815 participants (missing data among the two endogenous measures was estimated using Full Information Maximum Likelihood).

The sample consisted of 60% (*n* = 3,487) women and 40% (*n* = 2,328) men, with a mean age of 47.72 (*SD* = 15.60). With regard to education, 22.3% (*n* = 1,295) had no formal qualifications or did not report the qualification, 29.4% (*n* = 1,709) had some high school, 16.2% (*n* = 941) had a post-high school diploma or certificate, 23.0% (*n* = 1,335) had completed or were studying toward an undergraduate degree, and 9.2% (*n* = 535) had or were studying toward a post-graduate qualification. Participants' mean annual household income was $NZ 84,604 (*SD* = 70,629). Missing values for household income were replaced with the mean. To make the unstandardized slopes more interpretable, income was scaled in $10,000 units in the multilevel analyses.

In comparison to national census figures, the NZAVS data have overall more women and older, educated and wealthier respondents than the general New Zealand public [39]. These differences are common among samples taken from household surveys. Given that women, more educated, and wealthier individuals are generally more likely to be environmentally engaged [40]–[48], it is possible that our sample's responses on the outcome measures were higher than the general public's would be.

Political orientation was indexed by asking participants to rate their political orientation on a single-item scale from 1 (*extremely conservative*) to 7 (*extremely liberal*). Scores were reverse coded so that a higher score indicated greater political conservatism (*M* = 3.75, *SD* = 1.30).

#### Geographic and regional information

**Figure 1:**
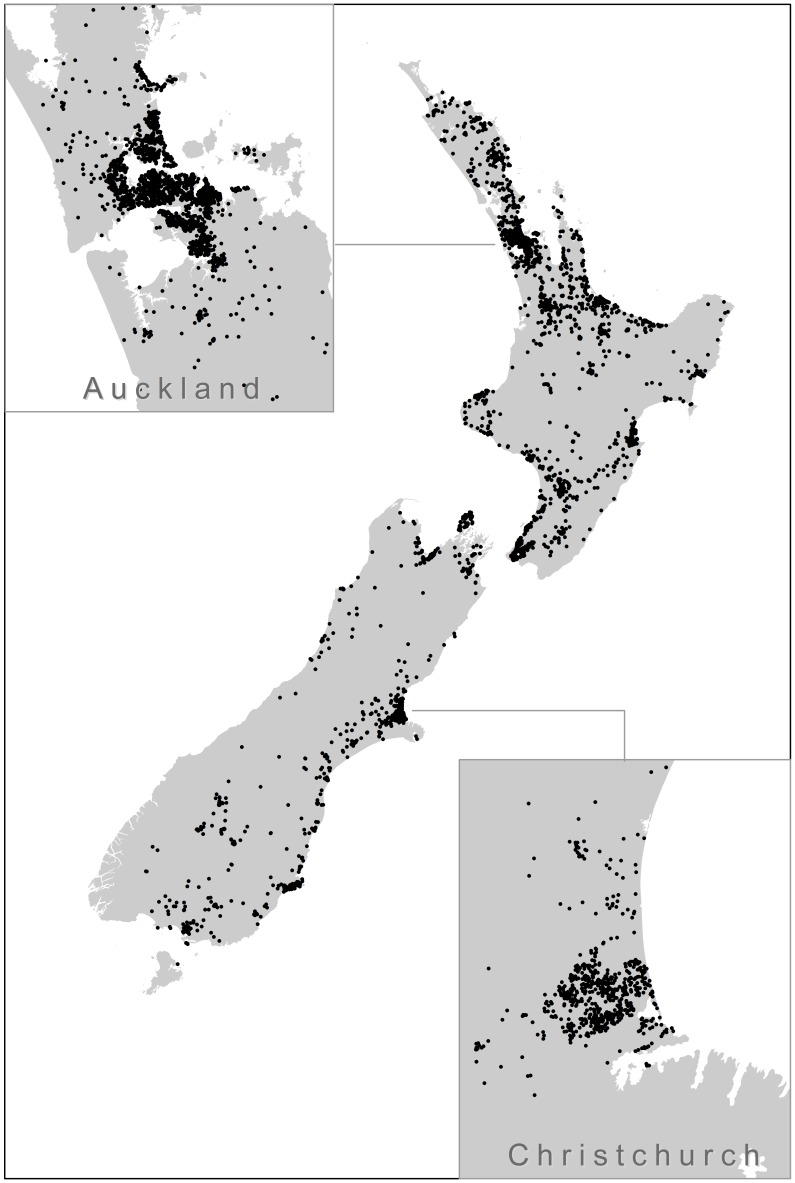
New Zealand map with geographic distribution of respondents. The figure provides inset maps with geographic distribution of respondents in the largest cities in the country.

Geographic and regional information was measured with 2006 New Zealand census data, based on the smallest geographical units (meshblock) in the census. Respondents provided their residential address, and this information was used to determine the meshblock boundaries within which each resided (see Figure 1). The geographic size of meshblocks is related to population density, but each unit tends to cover a region containing roughly 100 residents (*M* = 103, *SD* = 72, range = 3-1,431), and the average New Zealand meshblock size is about 9.66 km^2^ (3.73 mi^2^). Respondents in the present study (*N* = 5,815) were nested within 5,199 meshblocks, with a little over one person on average sampled per unit (*M* = 1.12, range 1–5).

We estimated the distance to the coast by calculating the straight-line distance (in kilometers) between the centroid of each meshblock and the nearest point on the shoreline (*M* = 9.87 km, *SD* = 17.01 km) using GIS analytic techniques. The distance was then assigned to all respondents living in the meshblock. Assigning respondents as if they all lived in the center of their meshblock is a reliable approximation because the majority of meshblocks are small [49]. We also estimated height above sea level for the centroid of each meshblock. To make the unstandardized slopes in our model more readily interpretable, distance to the coastline was scaled in 10 km units and height above sea level was scaled in meters.

#### Regional affluence

We also examined information about the net deprivation versus affluence of people residing in each meshblock. To measure deprivation we used the 2006 Deprivation Index, which provides detailed aggregate census information about the demographic characteristics of people residing in each area unit/neighborhood in New Zealand [50]. This index allocates a deprivation score to each meshblock based on the following nine variables in the census data (in weighted order): proportion of adults receiving a means-tested Government-supplied welfare benefit, household income, proportion not owning their own home, proportion of single-parent families, proportion unemployed, proportion lacking qualifications, proportion with household crowding, proportion with no telephone access, and proportion with no car access. The Deprivation Index thus reflects the average level of deprivation for neighborhoods (or small community areas) across the entire country. The Deprivation Index assigns a ranked decile score from 1 (most affluent) to 10 (most impoverished) to each geographical meshblock unit/neighborhood (*M* = 5.01, *SD* = 2.85).

#### Dependent measures: Climate change belief and support for emissions regulation

The two outcome measures were measured with single-item questions and were embedded in a larger battery of Likert-type questions rated on scale from 1 (*strongly disagree*) to 7 (*strongly agree*). Belief about the reality of climate change was assessed using the single item “Climate change is real” (*M* = 5.37, *SD* = 1.68). Support for government regulation of carbon emissions policy was assessed using the single item “The New Zealand government should be involved in regulating carbon emissions” (*M* = 4.84, *SD* = 1.68). Previous analyses of these two items focusing on their links with behavior intentions and overall distribution in the population have been reported elsewhere [2].

Note that these measures, like many psychological measures, are self-reported and therefore potentially subject to socially desirable responding, in which participants may exaggerate responses according to what they believe to be approved by others or what the researchers want to hear. However, this bias, especially for a household survey in which the researcher is not physically present, is unlikely to significantly affect results. Additionally, it is extremely unlikely that any participants guessed the purpose of the present study.

## Analysis

Multilevel modelling is recommended when there are two or more distinct, hierarchical levels of data [51]. With the present data, geographic location is one level and individuals another: One would expect that individuals from a given area (within-group variance) would have more similar characteristics than individuals from different areas (between-group variance). Multilevel analysis allows researchers to model the area-level and individual-level variance independently and simultaneously. We therefore constructed a multilevel model assessing the association between distance to the coast and both belief about the reality of climate change and support for government regulation of carbon emissions. These two outcomes were modelled simultaneously, and their Level 1 and Level 2 residual covariances were also included to adjust for residual associations. The intercepts for both outcomes were modelled as random effects, thus allowing the mean level of each outcome to vary across meshblocks.

We employed Maximum Likelihood with Robust estimation of the standard errors to adjust for possible non-normality of the residuals [51]. We first tested a model including only distance to the coast. We then tested a model that included sex, age, education, household income and political orientation at Level 1, and distance to the coast, height above sea level, the interaction between distance and height, and the level of economic deprivation of each meshblock at Level 2. This approach statistically adjusted for height above sea level, the interaction of height with distance, and the level of regional deprivation when assessing the association between distance to the coast and climate change attitudes, while also modelling the association between respondents' sex, age, education, political orientation and wealth on within-cluster variance. As noted above, studies have found that sex, age, education, political orientation, and wealth are all related to environmental concern [40]–[48]. Women are generally likely to be more concerned about the environment, as are younger, more educated, more liberal, and higher-income individuals.

Given the small number of residents sampled per meshblock, we modelled the slopes for Level 1 covariates as fixed effects. All Level 1 predictors were centered at the group mean. Level 2 predictors were centered at their grand mean.

## Results

Results from our multilevel random-intercept model are presented in Table 1. As predicted, distance from the coast significantly predicted decreased levels of belief in climate change (b = −.038, *p* = .004) and lower levels of support for government regulation of carbon emissions (b = −.036, *p* = .005). These unstandardized coefficients indicate that for every additional 10 km from the coast that residents live, they are likely to be .038 units lower in their belief in climate change, and .036 units lower in their support for government regulation of carbon emissions. Considering that ratings of the two outcomes ranged from a minimum possible value of 1 to a maximum of 7 (a total of 6 units), our model therefore indicates that someone who lives 100 km from the coastline will be on average .38 units lower in their belief in climate change [.38 = 10 * .038], or roughly 6.3% of the total possible scale range lower [6.33 = (.38/6) * 100], compared to someone who lives by the coastline. In New Zealand, the maximum distance from the coastline a person could reside is approximately 177 km. The effect of proximity is relatively small, but still statistically significant even considering the small geographical area of the country.

**Table 1:** Multilevel Random Coefficient Models assessing the associations between distance to the coast and climate change belief and support for government regulation of carbon emissions.

	b	se	t	p
***Model Predicting Climate Change Beliefs***				
**Step 1 Model**				
Constant	5.373	.021		
Distance to coast	−.038	.013	−2.89*	.004
**Step 2 Model**				
Constant	5.368	.023		
Distance to coast	−.054	.017	−3.11*	.002
Height above sea level	.001	.000	1.62	.105
Distance x Height	.000	.000	−.26	.799
Regional economic deprivation	.056	.008	7.26*	.000
Sex (being male)	−.426	.128	−3.32*	.001
Age	−.012	.005	−2.55*	.011
Education	.066	.055	1.19	.234
Household income	−.003	.009	−.27	.788
Political conservatism	−.148	.057	−2.57*	.010
***Model Predicting Support for Carbon Emissions Policy***				
**Step 1 Model**				
Constant	4.859	.021		
Distance to coast	−.036	.013	−2.83*	.005
**Step 2 Model**				
Constant	4.835	.024		
Distance to coast	−.047	.017	−2.81*	.005
Height above sea level	.000	.000	−.11	.911
Distance x Height	.000	.000	.84	.403
Regional economic deprivation	.037	.008	4.64*	.000
Sex (being male)	−.498	.122	−4.08*	.000
Age	.001	.004	.17	.862
Education	.075	.051	1.48	.138
Household income	−.021	.007	−2.79*	.005
Political conservatism	−.164	.056	−2.91*	.004

*Note*. Income was measured in units of $NZ 10,000. Distance from respondent address to nearest point on coast was scored in 10 km units. Height above sea level was scored in meter units. Distance to coastline, height above sea level, distance x height interaction, and regional deprivation were modelled at the between-region level, all other predictors were modelled at the within-region level.

These associations between distance to the coast and the outcome variables held in statistical significance and size in the extended model when also adjusting for the regional level of deprivation, height above sea level, and the interaction between distance and height. The model also included respondents' sex, age, education, household income and political orientation as predictors of the within-cluster variance in the outcome variables. Critically, this indicates that the focal association between proximity to the coast and the climate outcome variables was not moderated by height above sea level, and was not due to the fact that those in wealthier neighborhoods tend to live closer to the sea (shoreline areas in our sample were on average less deprived, *r* = −.04, *p*<.01) and tend to have a higher belief in climate change. Indeed, with regard to regional deprivation, our results showed the opposite pattern of effects. Respondents who lived in more deprived areas were more likely to both believe in climate change (b = .056, *p*<.001) and support government regulation of carbon emissions (b = .037, *p*<.001).

In terms of effects at the individual level (or Level 1 unit of analysis), education level and household income were not statistically associated with our outcome variables, while sex and political orientation exerted the strongest influence on both climate change belief and support for government regulation of carbon emissions. In accordance with previous findings [40]–[48], political conservatism predicted lower levels of belief in climate change (b = −.148, *p* = .010) and lower levels of support for government regulation of carbon emissions (b = −.164, *p* = .004), men showed decreased levels of belief in climate change (b = −.426, *p* = .001) and a lower level of support for government regulation of carbon emissions (b = −.498, *p*<.001), and age was significantly associated with climate change belief (b = −.012, *p* = .011), with older respondents tending to show less belief about the reality of climate change. The relative strength of the proximity effect (b = −.038 and b = −.036) was low in comparison with sex and political orientation, and it was slightly lower than the effect of regional deprivation, but our results indicate that distance to coast was significantly associated with climate change belief and support for emissions regulation.

## Discussion

In summary, proximity to the coast was associated with increased belief that climate change is real and increased support for government regulation of carbon emissions, irrespective of regional differences in affluence, residencies' average height above sea level, and individual differences in sex, age, education, political orientation, and wealth. These findings are in line with the other two studies showing that proximity to the coast is related to greater risk perception associated with global climate change [37] and with greater willingness to lower personal emissions [29]. Altogether, the findings demonstrate the importance of geographic location in engaging with the problem of climate change, expanding the profile of our understanding of climate-change-related perceptions, beliefs, and behaviors.

In all three studies, the findings indicate an association only; the studies were not able to discover the direction of the causal arrow. It may be that people with stronger belief in climate change tend to seek out coastal areas, or there might be some third variable (e.g., connectedness to nature) that causes both an increase in climate change belief and the tendency to reside in coastal areas. However, the theory of psychological distance [19], [21], [22] predicts that the experience or perception of greater climate change impacts, such as can be expected at the coasts [35], will bring the issue psychologically closer. This, in turn, is predicted to at least partially cause an increase in related variables such as belief in climate change.

Proximity to the coast certainly allows several possible ways in which belief in climate change could increase. For example, people residing at the coasts may be more likely to 1) experience large, climate-change-related impacts such as flooding and storms [31], [32], many of which are likely to have greater impact on coastal communities [35], 2) consider potential sea-level rise, which may require local adaptation [30], or 3) pay more attention to the local weather, as they plan to spend time outdoors at the beach. People residing at the coasts may also have an increased attunement to natural risk, since the ocean, as a large and constantly agitated body of water, may inspire a sense of respect for the power of nature and its changeability, and/or they may have an increased attunement to environmental problems of all types because such residents may care about the state of their beaches, pay attention to them, and have seen problems occur before. In sum, although the presented results are only an association, we note that such a causal relationship is consistent with psychological distance theory [19], [22].

Future research should seek to discover causality wherever possible. Longitudinal studies may be of interest; for example, researchers could examine climate-change-related variables across time among people who have moved closer to or more distant from the coast. Researchers could also consider including an item that specifically measures feelings of closeness or distance from climate change so that they can observe whether individual variables affect psychological distance in different ways. For example, weather-aware individuals may feel closer to climate change than others, but perhaps there is no difference between people who do or do not pay attention to the state of their beaches. Where possible, researchers should also specifically test for mediation by psychological distance. Statistical mediation tests the hypothesis that a mediator variable, such as psychological distance, is the intermediate cause between two other variables. For example, one could test the hypothesis that proximity to the coast causes an increase in psychological closeness to climate change, which in turn causes an increase in climate change belief.

There are, of course, other factors affecting climate change concern besides psychological distance. We have already mentioned that there are effects of socio-demographic variables such as age, sex, education, wealth, and political orientation [40]–[48], along with a variety of psychological barriers [16]–[20]. In addition, there is evidence that climate change concern is also affected by personality factors, such as individuals' traits and tendency to consider the potential future consequences of their actions [52]–[54], as well as one's trust in scientists [55] and (dis)belief in conspiracy theories [56].

Importantly, however, communicators are not in a position to change many of these other factors, such as personality factors and individual wealth. What the psychological distance evidence suggests, though, is that they may be able to create targeted campaigns—for example, discussing with individual metropolitan areas the effects they might expect to experience, and how to prepare for them. (This specific tactic has already been tested, resulting in an increase in willingness to reduce personal emissions [30].) Another tactic might include encouraging people to consider more deeply their experiences of local weather and environmental habitats, which may have already changed during their lifetimes—for example, unprecedented pine beetle outbreaks in North America have caused visible damage to forest areas [57]. Additionally, if the goal is to increase awareness of the risks in general, expending more energy on inland communities may be helpful, as it is clear that coastal communities are already slightly more risk-aware [37]. Ultimately, our findings support the view that psychological distance theory has important practical implications for climate change communication.
